# Decreased Interhemispheric Coordination in Treatment-Resistant Depression: A Resting-State fMRI Study

**DOI:** 10.1371/journal.pone.0071368

**Published:** 2013-08-02

**Authors:** Wenbin Guo, Feng Liu, Zhimin Xue, Keming Gao, Zhening Liu, Changqing Xiao, Huafu Chen, Jingping Zhao

**Affiliations:** 1 Mental Health Institute of the Second Xiangya Hospital, Key Laboratory of Psychiatry and Mental Health of Hunan Province, Central South University, Changsha, Hunan, China; 2 Mental Health Center, The First Affiliated Hospital, Guangxi Medical University, Nanning, Guangxi, China; 3 Key Laboratory for NeuroInformation of Ministry of Education, School of Life Science and Technology, University of Electronic Science and Technology of China, Chengdu, Sichuan, China; 4 Mood and Anxiety Clinic in the Mood Disorders Program of the Department of Psychiatry at Case Western Reserve University School of Medicine/University Hospitals Case Medical Center, Cleveland, Ohio, United States of America; 5 The Second Affiliated Hospital of Xinxiang Medical University, Henan Key Lab of Biological Psychiatry, Xinxiang Medical University, Xinxiang, Henan, China; University of Pennsylvania, United States of America

## Abstract

**Background:**

Previous studies have demonstrated that patients with treatment-resistant depression (TRD) and treatment-sensitive depression (TSD) differed at neural level. However, it remains unclear if these two subtypes of depression differ in the interhemispheric coordination. This study was undertaken for two purposes: (1) to explore the differences in interhemispheric coordination between these two subtypes by using the voxel-mirrored homotopic connectivity (VMHC) method; and (2) to determine if the difference of interhemispheric coordination can be used as a biomarker(s) to differentiate TRD from both TSD and healthy subjects (HS).

**Methods:**

Twenty-three patients with TRD, 22 with TSD, and 19 HS participated in the study. Data of these participants were analyzed with the VMHC and seed-based functional connectivity (FC) approaches.

**Results:**

Compared to the TSD group, the TRD group showed significantly lower VMHC values in the calcarine cortex, fusiform gyrus, hippocampus, superior temporal gyrus, middle cingulum, and precentral gyrus. Lower VMHC values were also observed in the TRD group in the calcarine cortex relative to the HS group. However, the TSD group had no significant change in VMHC value in any brain region compared to the HS group. Receiver operating characteristic curves (ROC) analysis revealed that the VMHC values in the calcarine cortex had discriminatory function distinguishing patients with TRD from patients with TSD as well as those participants in the HS group.

**Conclusions:**

Lower VMHC values of patients with TRD relative to those with TSD and those in the HS group in the calcarine cortex appeared to be a unique feature for patients with TRD and it may be used as an imaging biomarker to separate patients with TRD from those with TSD or HS.

## Introduction

Despite the rapid progress that has been made in the development of antidepressants over the years, approximately one-third of depressed patients still fail to respond to standard antidepressant treatments [1]. Patients who do not respond to a series of standard treatments are classified as having treatment-resistant depression (TRD), while those who respond to standard treatment are classified as having treatment-sensitive depression (TSD) [2]. Treating TRD remains a common therapeutic challenge for psychiatrists [3], [4]. To overcome this challenge is to develop more effective treatment for major depressive disorder (MDD), especially TRD. This effort requires better understanding of the pathogenesis of MDD [5].

As a part of the efforts to understand the pathogenesis of MDD, the structural and functional changes associated with MDD have been explored with imaging technologies. A bulk of positron emission tomography (PET) and functional magnetic resonance imaging (fMRI) studies have accumulated enough evidence to suggest that MDD is related to widespread local changes in many brain areas within the cortico-limbic circuit (See L et al. [6] for a review). In addition, electroencephalography (EEG) studies consistently reveal frontal alpha asymmetry in patients with MDD [7] which predicted treatment response in depression [8]. Several studies have shown that positive emotions are related to greater relative activity in left frontal area; whereas negative emotions are related to higher relative activity in right frontal area [9], [10]. Therefore, frontal hemispheric difference in EEG activity may be a characteristic presentation of patients with depression.

Moreover, MDD has been increasingly conceptualized as a disorder of brain disconnectivity. Abnormality of the limbic-cortical networks is speculated to play a key role in the pathogenesis of MDD. This speculation is supported by the findings of functional connectivity (FC) studies. For example, several studies revealed that patients with MDD had alterations of orbitofrontal cortex-precuneus coupling [11] and pregenual anterior cingulate cortex (ACC)-dorsomedial thalamus coupling [12]. Other studies have shown bilateral alterations in the prefrontal regions as well as the temporal limbic regions [13]. However, there has never been a study directly exploring TRD-related alterations in functional interhemispheric coordination between the cerebral hemispheres.

Functional homotopy, the synchrony in patterns of spontaneous activity between homotopic regions in each brain hemisphere, is one of the most salient aspects of the brain’s intrinsic functional architecture [14]. The degree of correlated activity between homotopic interhemispheric counterparts may represent the importance of interhemispheric communication to integrate brain function for underlying coherent cognition and behavior [15]. As mentioned above, the vast majority of task-based and resting-state fMRI (R-fMRI) studies in MDD suggest that the affected brain regions are bihemispheric [16]. Previously, by using a 3T Siemens version scanner, we found that homotopic resting-state FC was disrupted in first-episode, drug-naive MDD [17]. Based on these findings, homotopic resting-state FC studies may offer an opportunity to investigate regional function alterations to determine whether the change in functional homotopy can be used to distinguish TRD from both TSD and HS or distinguish TSD from HS.

Patients with TRD and TSD respond to antidepressants differently, suggesting that the two subtypes of depression differ at the neural level. In a seed-based FC study, TRD was related to disrupted FC mainly in thalamo-cortical circuits, while non-treatment refractory depression was associated with more distributed decreased FC in the limbic-striatal-pallidal-thalamic circuits [18]. Recently, using the coherence-based regional homogeneity (Cohe-ReHo) method, we detected differences between the two subtypes in regional alterations in the cerebellum [19]. These data further support that the two subtypes of MDD differ at the neural level. However, it remains equivocal if the two subtypes of depression differ in interhemispheric coordination.

To determine if there is any difference in interhemispheric coordination between patients with two types of depression as well as between depressed patients and healthy subjects, we analyzed an existing dataset [19] with voxel-mirrored homotopic connectivity (VMHC) method. The VMHC is a novel approach which directly compares the interhemispheric resting-state FC [20], and has been used in studying different psychiatric conditions [15], [21]. The VMHC quantifies the resting-state FC between each voxel in one hemisphere and its mirrored voxel in the opposite hemisphere. Based on the facts that: (1) patients with TRD or TSD respond to antidepressants differently; and (2) both groups of patients had disruption in different brain circuits [18], we hypothesized that the two patient groups would exhibit reduced VMHC in different brain regions. Given that frontal dysfunction is associated with MDD [11], [22]–[24], we expected that the frontal lobe would be particularly affected. We also hypothesized that the detected changes in VMHC could be utilized as biomarkers to separate patients with TRD from those with TSD and from healthy subjects (HS).

## Methods

### Subjects

Twenty-four patients with TRD, 31 with first-episode, treatment-naive MDD were recruited from the Mental Health Institute of the Second Xiangya Hospital, Key Laboratory of Psychiatry and Mental Health of Hunan Province, Central South University, Hunan, China. The subjects in the present study were from one of our previous studies [19]. Among the 27 female patients, 13 (6 patients with TRD and 7 patients with treatment-naive MDD) were maternal, but none had postpartum depression.

In order to be eligible for this present study, all patients had to meet the following inclusion criteria: (1) In a current major depressive episode diagnosed with a Structured Clinical Interview for DSM-IV (SCID) [25]; (2) Within 18–50 years of age; (3) Right-handed Han Chinese; and (4) HRSD score of ≥18.

All patients with TRD were in a treatment-resistant state and had taken at least two classes of antidepressants before participating in the study. For patients with TRD there was a one week drug-free period prior to the MRI scan. This one week wash-out period was selected based on the fact that the half-life of most antidepressants (except for fluoxetine) is from 12- to 24-hours. Treatment resistance was defined as non-responsiveness to at least two adequate treatments with adequate dosage and duration (at least 6 weeks for each trial), compliance, and use of different types of antidepressants [26]. Non-responsiveness was defined as a reduction of <50% in the 17-item Hamilton Rating Scale for Depression (HRSD) [27] after the treatment with a minimum dose of 150 mg/day of imipramine equivalents for 6 weeks [28]. Detailed treatments and other clinical characters of patients with TRD are exhibited in Table S1 and S2.

The treatment-naive patients were directed to take an antidepressant at a minimum dose of 150 mg/day of imipramine equivalents for 6 weeks after the MRI scan. The antidepressant included one of the three typical classes: tricyclic antidepressants (TCAs), selective serotonin reuptake inhibitors (SSRIs), and serotonin-norepinephrine reuptake inhibitors (SNRIs). Treatment response was defined as more than a 50% reduction in the HRSD scores after treatment [26]. Only patients showing response after 6-week treatment were enrolled in the study as patients with TSD.

Twenty HS were recruited from the community. They were matched with the patient groups in age, gender, and education level. Patients or HS were excluded if they had any of the following: (1) Any history of neurological diseases, other medical illnesses, or other psychiatric disorders such as schizophrenia, bipolar disorders, or substance-induced mood disorder; (2) Any current Axis I comorbidities such as anxiety disorders, alcohol or drug dependence; (3) Any severe Axis II personality disorders or mental retardation; and (4) Any contraindication for MRI.

The study was approved by the Ethics Committee of the Second Xiangya Hospital. All participants were given information about the procedures and signed an informed consent document.

### Image Acquisition

Scanning took place on a 1.5T GE scanner (General Electric, Fairfield, Connecticut, USA) equipped with high-speed gradients at the recruitment day. Participants were instructed to lie motionless, close their eyes and remain awake. The following parameters were used to obtain functional images: repetition time/echo time (TR/TE) = 2000/40 ms, 20 slices, 64×64 matrix, 90° flip angle, 24cm FOV, 5 mm slice thickness, 1 mm gap, and 180 volumes (6 min).

### Data Preprocessing

Resting-state data were preprocessed in Matlab using the statistical parametric mapping software package (SPM8, http://www.fil.ion.ucl.ac.uk/spm). The first 10 volumes were discarded to ensure a steady state condition. Following steps including slice timing, head motion correction, and spatial normalization (voxel size: 3×3×3 mm^3^) were performed using Data Processing Assistant for Resting-State fMRI (DPARSF) [29]. The processed images were smoothed with an isotropic Gaussian kernel (full-width at half-maximum: 8 mm). Linearly trend removing and band-pass (0.01–0.08 Hz) filtering were conducted using REST [30]. Several sources of spurious covariates and their temporal derivatives were then removed using linear regression. These covariates included six head motion parameters obtained by rigid body correction, the signal from a ventricular region of interest (ROI), and the signal from a region centered in the white matter [31].

### Interhemispheric Correlation

The VMHC was processed with software REST. Briefly, Pearson correlations (Fisher *z*-transformed) between a given voxel and a mirrored voxel in the opposite hemisphere were computed to generate VMHC maps. The details of VMHC obtainment were described in a previous study [20].

Voxel-based comparisons of the entire VMHC maps with ANOVA were conducted in REST with a significance threshold of *p*<0.005 for multiple comparisons using Gaussian Random Field (GRF) theory (min *z*>2.807, cluster significance: *p*<0.005, corrected). Post-hoc t-tests, restricted to the voxels showing significant differences by ANOVA analyses, were performed to identify differences between each pair of groups. The resulting statistical map was set at *p*<0.005 (GRF corrected). Since small amount of head motion can influence resting-state FC results [32], we computed framewise displacement (FD) for our data, which provided the temporal derivative of the motion parameters. These FD parameters were applied as covariates at the group level comparisons.

Brain regions exhibiting significant differences between the TRD and TSD groups and between the TRD and HS groups were designed as masks. Mean VMHC values were extracted within the masks for further receiver operating characteristic curves (ROC) analysis.

To examine the association of VMHC and clinical variables such as HRSD scores and illness duration, voxel-based correlations were implemented to the VMHC values of each depressed patient and clinical variables for each depressed group separately and together (*p*<0.005, GRF corrected).

### Seed-based FC

Seed-based FC was performed using a temporal correlation approach [33]. Brain regions showing significant differences in VMHC maps between the TRD and TSD groups and between the TRD and HS groups were defined as seed ROIs. Correlation analyses were conducted between the seeds and the remaining voxels. The resulting *r* values were transformed to *z* values to improve the Gaussianity of their distribution. For each group and each seed, individual *z* maps were compared with a random effect one-sample *t* test to detect voxels showing significant correlations with the seeds (*p*<0.005, GRF correction). Afterwards, voxel-based comparisons of the entire brain *z* maps with ANOVA were conducted in REST by using an explicit mask from the union set of the one-sample *t*-test results (*p*<0.005, GRF correction). Then post-hoc *t*-tests were performed to identify differences between each pair of groups (*p*<0.005, GRF correction). The FD of each subject was used as a covariate at the group level analyses.

## Results

### Subjects

Data from four subjects (1 TRD, 2 TSD, and 1 HS) were excluded for further analyses due to excessive head motion. Data from 7 treatment-naive patients were discarded due to treatment non-response. Finally, the participants included 23 patients with TRD, 22 patients with TSD and 19 HS. Demographics and clinical characteristics of these participants are presented in Table 1. The three groups did not differ significantly in age, gender, education level, and the FD parameter. There was no significant difference in HRSD total scores and the scores of HRSD subscales between the patient groups. However, the TRD group had longer overall illness duration as well as current illness duration than those of the TSD group.

**Table 1:** Characteristics of patients with TRD or TSD and HS.

Variables(Mean±SD)	TRD	TSD	HS	*P* value
N (M/F)	23(11/12)	22(12/10)	19(10/9)	0.898^a^
Age, years	27.35±7.26	28.09±9.91	24.37±4.18	0.269^b^
Education, years	13.13±3.44	12.23±2.62	13.11±2.47	0.510^b^
Mean displacement (mm)	0.09±0.04	0.09±0.03	0.10±0.04	0.468^b^
Overall illness duration, months	27.43±35.89	2.95±1.73		0.004^c^
Current illness duration, months	12.22±6.08	2.95±1.73		<0.001^c^
HRSD score	24.52±4.17	25.89±6.26		0.407^c^
Factors of HRSD				
1 Anxiety	6.96±1.89	7.78±2.16		0.202^c^
2 Weight loss	0.57±0.90	0.94±0.94		0.195^c^
3 Cognitive disturbance	5.39±1.70	6.28±1.93		0.127^c^
4 Retardation	7.96±1.52	7.67±1.94		0.595^c^
5 Sleep disturbance	3.65±2.01	3.17±1.47		0.395^c^

a The *P* value for gender distribution in the three groups was obtained by chi-square test.

b The *P* values were obtained by one-way analysis of variance tests.

c The *P* values were obtained by two sample *t*-test.

TRD = treatment-resistant depression.

TSD = treatment-sensitive depression.

HS = healthy subjects.

HRSD = Hamilton Rating Scale for Depression.

### VMHC: Group Differences

**Figure 1:**
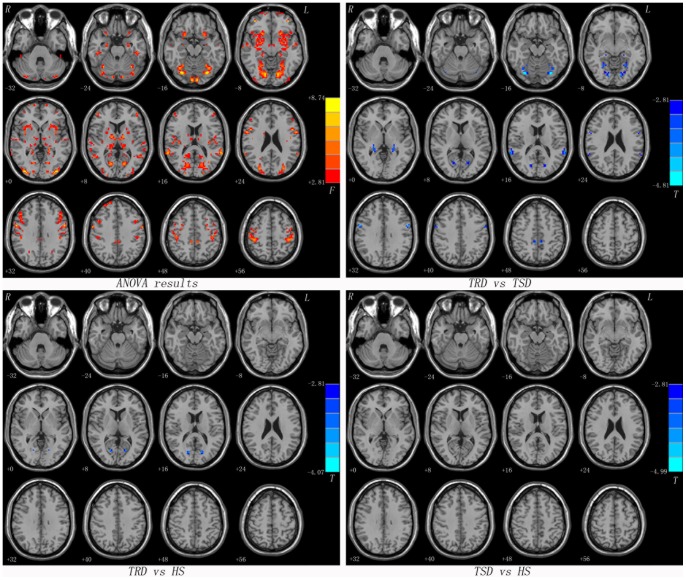
Statistical maps showing VMHC differences in different brain regions between studied groups. Red denotes VMHC differences in the ANOVA analyses and the yellow/red color bar indicates the *F* value from the ANOVA analyses. Blue denotes lower VMHC and the green/blue color bars indicate the *T* value from post hoc analysis between compared groups. Of note, the two-sample t-test results within a mask showed significant group differences in the ANOVA analysis. VMHC = voxel-mirrored homotopic connectivity, TRD = treatment-resistant depression, TSD = treatment-sensitive depression, HS = healthy subjects.

As shown in Figure 1 and Table 2, significant differences between TRD and TSD in the VMHC values were observed throughout the cerebellum, the frontal, temporal, parietal, occipital lobes and subcortical or limbic regions by ANOVA. Compared to the TSD group, the TRD group showed significantly lower VMHC values in the calcarine cortex, fusiform gyrus, hippocampus, superior temporal gyrus, middle cingulum and precentral gyrus. Besides, lower VMHC values were observed in the TRD group in the calcarine cortex relative to the participants in the HS group. However, there was no significant difference in the VMHC values in any brain region between the TSD group and the HS group. In addition, the pooled patients (TRD and TSD) exhibited lower VMHC in the postcentral gyrus compared to the HS group (Table S3).

**Table 2:** Significant differences in VMHC values between groups.

Cluster location	Peak (MNI)			Cluster size	*T* value
	x	y	z		
TRD<TSD					
Calcarine Cortex	±18	–69	6	27	−3.9644
Fusiform Gyrus	±33	−75	−15	81	−4.8120
Precentral Gyrus	±57	0	33	31	−4.4508
Hippocampus	±24	−33	−3	18	−4.0928
Superior Temporal Gyrus	±63	−45	12	31	−3.8810
Middle Cingulum	±9	−39	45	22	−4.1867
TRD<HS					
Calcarine Cortex	±15	−72	12	29	−3.8629
TSD<HS					
None					

VMHC = voxel-mirrored homotopic connectivity.

TRD = treatment-resistant depression.

TSD = treatment-sensitive depression.

HS = healthy subjects.

### Seed-based FC

**Figure 2:**
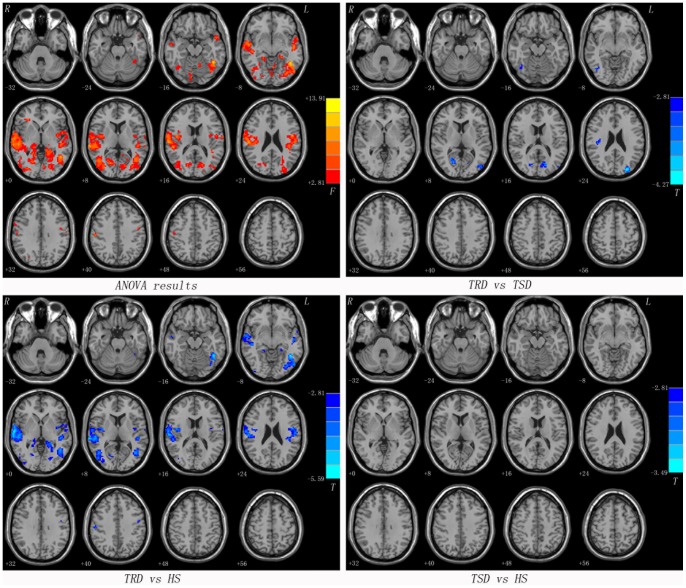
Statistical maps showing seed-based FC differences between subject groups (seed: the left calcarine cortex). Red denotes FC differences in the ANOVA analyses and the color bar indicate the *F* value from the ANOVA analyses. Blue denotes lower FC and the color bars indicate the *T* value from post hoc analysis between each pair of groups. Of note, the two-sample *t*-test results within a mask showed significant group differences in the ANOVA analysis. FC = functional connectivity, TRD = treatment-resistant depression, TSD = treatment-sensitive depression, HS = healthy subjects.

**Figure 3:**
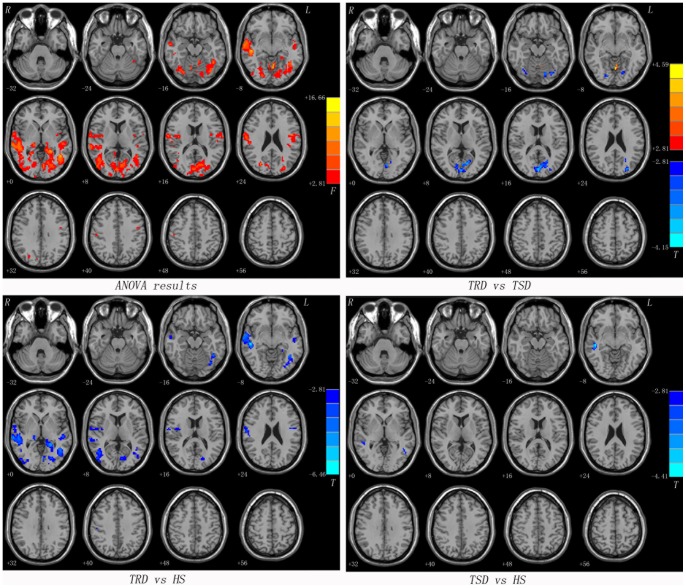
Statistical maps showing seed-based FC differences between subject groups (seed: the right calcarine cortex). Red denotes FC differences in the ANOVA analyses and the color bar indicate the *F* value from the ANOVA analyses. Red and blue denote higher and lower FC respectively and the color bars indicate the *T* value from post hoc analysis between each pair of groups. Of note, the two-sample *t*-test results within a mask showed significant group differences in the ANOVA analysis. FC = functional connectivity, TRD = treatment-resistant depression, TSD = treatment-sensitive depression, HS = healthy subjects.

As mentioned above, the calcarine cortex exhibited lower VMHC values in the TRD group compared to both the TSD group and the HS group. We examined whole-brain resting-state FC associated with two ROIs (one calcarine cortex per hemisphere). Most of seed ROIs showed altered FC within the brain regions of the posterior perceptual and motor areas (such as the middle temporal gyrus, the middle occipital gyrus, the precentral gyrus and the cerebellum) along with a few subcortical regions (such as the thalamus) (Figure 2, 3 and Table 3).

**Table 3:** Significant differences in seed-based resting-state FC between groups.

Seeds	Connectedregions	Peak (MNI)			Clustersize	*T* value
		x	y	z		
*TRD>TSD*						
R Calcarine	Vermis 6	0	−63	−9	22	4.5880
*TRD<TSD*						
L Calcarine	R Inferior TemporalGyrus	45	−66	−12	29	−3.7247
	R Calcarine	21	−66	6	56	−4.2185
	L Middle OccipitalGyrus	−45	−81	12	28	−4.1227
	L Cuneus	−18	−78	15	33	−3.5581
	R Insula	42	−24	24	27	−3.4162
	L Middle OccipitalGyrus	−27	−87	24	45	−4.2684
R Calcarine	L Lingual Gyrus	−18	−75	−12	51	−3.7115
	R Lingual Gyrus	18	−84	−12	20	−3.9199
	L/R Calcarine	0	−87	12	213	−4.1546
	L Middle OccipitalGyrus	−27	−84	24	41	−3.6235
*TRD<HS*						
L Calcarine	L Inferior TemporalGyrus	−45	−51	−12	409	−5.5896
	R Lingual Gyrus	15	−39	−3	38	−3.4187
	R Postcentral Gyrus	60	−21	18	846	−5.5417
	L Superior TemporalGyrus	−45	−30	3	112	−4.3217
	L Precuneus	−24	−45	0	66	−3.9812
	R Calcarine	21	−66	3	29	−3.6190
	R Middle TemporalGyrus	45	−63	9	152	−4.4457
	L Superior Temporal Gyrus	−60	−12	3	39	−3.3182
	L Precentral Gyrus	−48	−6	24	157	−4.2126
	R Postcentral Gyrus	51	−24	42	20	−4.4052
R Calcarine	L Middle TemporalGyrus	−45	−54	−3	398	−4.9090
	R Middle TemporalGyrus	51	−27	−6	519	−6.4587
	L Middle TemporalGyrus	−54	−12	−12	59	−4.1010
	L Lingual Gyrus	−24	−45	0	92	−5.1995
	R Middle OccipitalGyrus	48	−75	6	165	−4.0451
	R Thalamus	15	−27	−3	19	−3.7144
	L Precentral Gyrus	−48	−6	24	20	−3.6062
	R Postcentral Gyrus	51	−21	42	16	−4.0321
*TSD<HS*						
R Calcarine	R Middle TemporalGyrus	48	−24	−9	54	−4.4069
	L Middle TemporalGyrus	−48	−54	−3	15	−3.4295

FC = functional connectivity; MNI = Montreal Neurological Institute; TRD = treatment-resistant depression; TSD = treatment-sensitive depression; HS = healthy subjects; R = right; L = left.

### ROC Analysis between the Patient Groups

**Figure 4:**
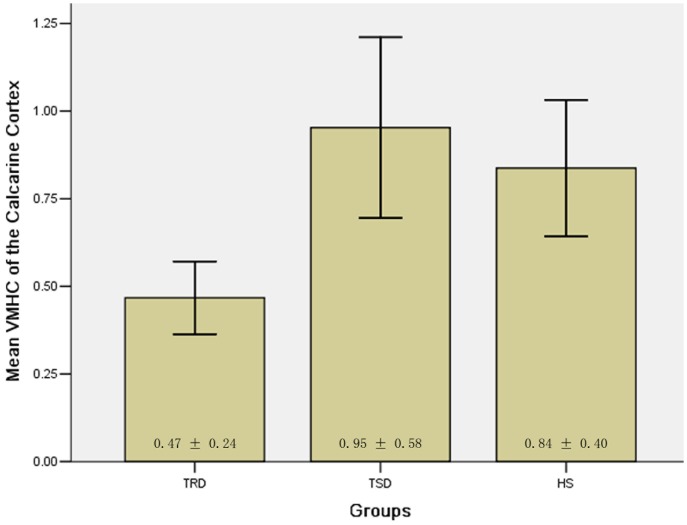
Bar plots representing the mean (and standard error) VMHC value of the calcarine cortex. Significant differences found between the TRD group and the TSD group or the HS group (TRD vs TSD: *p*<0.001; TRD vs HS: *p*<0.01). VMHC = voxel-mirrored homotopic connectivity, TRD = treatment-resistant depression, TSD = treatment-sensitive depression, HS = healthy subjects.

**Figure 5:**
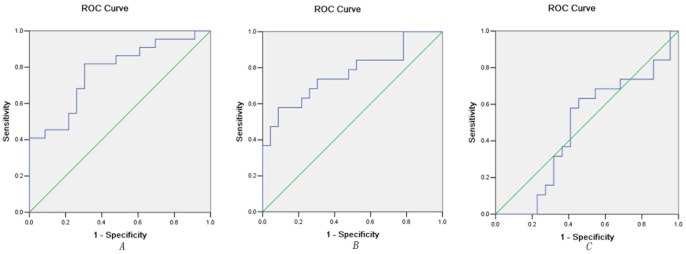
Receiver operating characteristic (ROC) curves by using the mean VMHC value in the calcarine cortex. A. Separating TRD from TSD; B. Separating TRD from HS; C. Separating TSD from HS. VMHC = voxel-mirrored homotopic connectivity, TRD = treatment-resistant depression, TSD = treatment-sensitive depression, HS = healthy subjects.

Since the calcarine cortex exhibited significant VMHC differences between the patient groups and between patients with TRD and the HS group, it might be used as a biomarker to separate the patients with TRD from those with TSD and from HS. To test this possibility, mean VMHC values were extracted from the calcarine cortex (Figure 4) and ROC analysis was conducted. The areas under the curves of the calcarine cortex were relatively high. Further diagnostic analysis revealed that the sensitivity and specificity of separating TRD from TSD and from HS were relatively high (Figure 5 and Table 4).

**Table 4:** ROC analysis for differentiating different studied groups.

Brain regions	Area Under the Curve	Cut-off point	Sensitivity	Specificity
*Separating TRD from TSD*				
Calcarine Cortex	0.777	0.5497^a^	73.91%(17/23)	81.82%(18/22)
*Separating TRD from HS*				
Calcarine Cortex	0.769	0.7823	91.30%(21/23)	68.42%(13/19)
*Separating TSD from HS*				
Calcarine Cortex	0.476	0.7498	54.55%(12/22)	63.16%(12/19)

a. By this cut-off point, the VMHC value of calcarine could correctly classify 17 of 23 patients with TRD and 18 of 22 patients with TSD, resulted in a sensitivity of 73.91% and a specificity of 81.82%. The means of other cut-off points were similar. VMHC = voxel-mirrored homotopic connectivity, TRD = treatment-resistant depression, TSD = treatment-sensitive depression, HS = healthy subjects.

### Voxel-based Correlations between VMHC and Clinical Variables

No correlation was found between VMHC in any brain region and HRSD scores in each patient group and the combined patient group by voxel-based correlation analyses. There was also no correlation between VMHC and age, education level, the overall illness duration, or the current illness duration.

## Discussion

To our knowledge, this is the first study of using the VMHC to explore interhemispheric resting-state FC in patients with TRD or TSD and healthy controls. We found that patients with TRD had lower VMHC values in the calcarine cortex compared to both TSD and HS groups. However, there was no significant difference between TSD and HS in VMHC values in the calcarine cortex.

The calcarine cortex is where the primary visual cortex is located and plays an important role in “refocusing” of attention [34]. Lower VMHC in this region may disrupt the lower-level function of attentional control. Normally, the fronto-parietal cortex is the primary source of top-down attentional control signals [35]. The modulation of visual input with attentional engagement follows as a consequence of top-down control processes [36]. In addition to direct interhemispheric interaction, lower-level areas like the calcarine cortex may act by sending projections to the high-level areas, such as the prefrontal cortex (PFC). Thereafter, reduced VMHC in this area in patients with TRD will interfere with the function of high-level areas and lead to a top-down disconnection. This kind of top-down disconnection is supported at least by one fMRI study showing that attentional shifts generate activity not only within the fronto-parietal cortex but also within the calcarine cortex [34].

Reduced VMHC values in other brain regions in patients with TRD compared to patients with TSD suggest that the disconnection of other brain regions may play a role in the pathogenesis of TRD. The fusiform gyrus is involved in the perception of facial emotion. The impairment of the fusiform gyrus has been observed in MDD [37]. As a core region of the limbic system, the hippocampus is involved not only in learning and memory [38] but also in the regulation of motivation and emotion [39]. The superior temporal gyrus plays a distinct role in emotional processing and social cognition [40]. Moreover, hypoactivity of the middle cingulum and precentral gyrus was associated with executive dysfunction and psychomotor symptoms in MDD [41]. Reduced VMHC in these regions may be the neuronal basis of depressive symptoms of TRD. Since the reduced VMHC values in these regions were only found between TRD and TSD, but not between TRD and HS or between TSD and HS, they might be state-related changes in the TRD group.

The insignificant difference between TSD and HS groups in the VMHC values is somewhat unexpected. There are a few possibilities. First is the duration of illness. The TSD group included young adults with short overall/current illness duration (mean: 2.95 months), first-episode, and drug-naive MDD. The short duration of the illness may be responsible for the insignificant VMHC change in the TSD group. Second is the sensitivity of scanners. Using a 3T Siemens version scanner, we found that patients with first-episode and drug-naive MDD had reduced VMHC values in the medial prefrontal cortex (MPFC) and the posterior cingulate cortex/precuneus (PCC/PCu) [17]. The TSD patients in the current study were similar to those in the previous study [17]. Since the original data from these two studies were obtained from different scanners, we can not make a direct group comparison. However, the results of the current study suggest that the VMHC method used in the current study might not be sensitive enough to detect VMHC change in TSD patients. Third is the statistical power. When we loosed our statistical power to uncorrected voxel-wise threshold of *p*<0.005, postcentral gyrus also exhibited significantly decreased VMHC values (Table S4). These data suggest that disrupted functional homotopy in certain brain areas may also attribute to the pathogenesis of TSD.

There have been reports that the PFC acts in a key role with both cognition and emotion. Abnormal activity in the PFC has been observed in patients with MDD by using both R-fMRI [42]–[44] and task-based fMRI methods [45]. These studies suggest that PFC changes are a consistent feature of depressed patients. In light of these studies, the lack of the PFC changes in both the TRD and TSD groups in the present study is somewhat unexpected. In addition to factors such as depression severity, heterogeneity of subjects, and medication treatments, there are at least two other possibilities which might account for this inconsistency. First, the inconsistency could be due to different analytic methods used in different studies. The VMHC is designed to evaluate the interhemispheric resting-state FC. It is possible that the lower-level perceptual and motor areas send unilateral projections to the high-level areas (such as the PFC) in addition to projecting directly to both hemispheres in order to interhemispherically execute their influences [46]. Thus, reduced VMHC in the lower-level areas could contribute to the deficits in the high-level areas although impaired interhemispheric interaction can not be discovered in these areas. This assumption is partly supported by our previous Cohe-ReHo analysis, which showed that the patients in the current study exhibited lower Cohe-ReHo in the bilateral superior frontal gyrus [19]. The Cohe-ReHo method was developed to measure the local signal coherence or synchronization within a functional cluster (usually 27 voxels) [47]. Evidence from functional neuroimaging studies on humans suggests that top-down attentional control processes encompass the superior frontal, inferior parietal and superior temporal cortex [36]. Combined with the previous results, the present results support the idea that the lower-level areas (such as the calcarine cortex in the present study) send unilateral projections to the high-level areas (such as the PFC) in addition to direct interhemispherically mediated influences [46].

Another possibility is the relatively strict corrected *p* value adopted in the present study. When we loosed our statistical power to uncorrected voxel-wise threshold of *p*<0.005, some PFC areas also exhibited significantly decreases in VMHC values (Table S4). This phenomenon implies that disrupted functional homotopy in the PFC areas may also attribute to the pathogenesis of MDD to some extent.

The VMHC approach adopted here to identify brain regions with abnormal interhemispheric FC has proved to be informative. However, the mechanism behind these deficits in VMHC remains equivocal. There are several possibilities for the deficits in VMHC. First, white matter deficits in the corpus callosum could disrupt the synchrony between mirrored connected regions because neural signals are not transmitted with fidelity. However, our previous analyses using the tract-based spatial statistics (TBSS) method in the same group of patients with TRD or TSD did not show any white matter alteration between the mirrored regions with decreased VMHC [48], [49]. In addition, no direct relationship between VMHC and white matter diffusion was observed previously [15], [21]. These findings imply that structural and functional metrics estimate different aspects of interhemispheric connectivity.

Second, gray matter dysfunctions might account for the deficits in VMHC. In the intrinsic neural activity, the correlation of fMRI signals of gray matter is an indirect index of synchrony [50], [51]. Similar to interhemispheric coherence, the correlation of fMRI signals between homotopically connected brain regions has been employed to measure the strength of resting-state FC [21]. The findings indicate that normal interhemispheric FC may arise from more complex pathways, such as common subcortical drivers or complex network-level synchronization, neither of which needs direct structural connectivity between cortical counterparts. VMHC is one of the most salient aspects of the brain intrinsic functional architecture [14], [16], which possibly represents the importance of interhemispheric communication to integrate brain function underlying coherent cognition and behavior [15]. Clinically, the disruption in VMHC has been reported in schizophrenia [46], depression [17], autism [21] and cocaine addiction [15].

Since epidemiological studies have shown that women have a higher risk for developing MDD than men, gender difference in brain structure and function has attracted researchers’ attention. However, the gender difference in imaging data is far from settled [52]. Some researchers have shown that there was no gender difference in whole gray matter volume, and the activity of the frontal lobe and the temporal lobe when the brain volume was controlled [53]. However, smaller brain volume has been observed in females than in males. Females had a higher percentage of gray matter, but males had a higher percentage of white matter [54]. It would be useful to conduct an fMRI study between males and females with different stages of MDD. The sample sizes in the current study are not large enough to address gender specific questions. Larger sample studies comparing gender difference in brain functioning in patients at different stages of MDD are warranted.

Since clinical variables such as the duration and severity of a depressive episode have been reported as predictors for treatment non-response [55]–[58], the finding in the present study of no correlation between the decreased VMHC values in the calcarine cortex and these variables was somewhat unexpected. Although this finding could be confounded by the small sample size of our study, it is also possible that the alterations of VMHC in the calcarine cortex may be a trait marker for patients with TRD independent of the severity of symptoms and other related factors.

In addition to the relatively small sample size, the present study has several other limitations. First, patients with TRD were in the current treatment-resistant state and had tried at least two types of antidepressants before the recruitment. Previous studies have shown that antidepressants made brain activity of depressed patients more similar to that of healthy subjects [59], [60]. Hence, the current findings in the TRD group were more likely due to the nature of the illness rather than the effects of the drugs. Second, the effect of the overall/current illness duration on the disorder remains to be debated. The TRD group had longer overall illness duration as well as longer current illness duration than those of the TSD group. The two variables were not used as covariates in the group comparisons for the reason that illness chronicity is an inherent characteristic of the TRD group and can not be removed [61]. However, no correlation was found between the VMHC in any brain region and the overall illness duration or the current illness duration by voxel-based correlation analyses. Third, cortical structures are not completely symmetrical. Using a symmetrical standard template in the current study might have biased the final results. Finally, global signal regression (GSReg) can bias correlations and complicate the explanation of negative correlations. The GSReg can also introduce a regionally varying correlation bias that is dependent on the unknown true underlying correlation structure [62]. Therefore, the GSReg was not utilized in the present study. It is unknown whether the current findings have been biased by the global signal without GRReg since the GSReg has distorting effect on interregional correlations.

In conclusion, the decreased VMHC values in the calcarine cortex may represent an imaging biomarker for TRD and may be used to separate patients with TRD from those with TSD as well as from HS. The findings also suggest that VMHC may offer an important new avenue of exploring the homotopic FC in both TRD and TSD in order to better understand the nature of the deficits in patients with MDD.

## Supporting Information

Table S1 Current treatment details of patients with TRD.
(DOC)

Table S2 The number of episodes of patients with TRD.
(DOC)

Table S3 Significant VMHC differences between pooled patients and HS (*p*<0.005, GRF correction).
(DOC)

Table S4 Significant VMHC differences between groups (uncorrected voxel-wise threshold of *p*<0.005).
(DOC)
