# Supplementary material for: Decreased Interhemispheric Coordination in Treatment-Resistant Depression: A Resting-State fMRI Study
Source: PLoS One. 2013 Aug 2;8(8):e71368. doi: 10.1371/journal.pone.0071368 (PMC3732240; doi:10.1371/journal.pone.0071368)
Supplement: Table S1 — Current treatment details of patients with TRD. (DOC) [file pone.0071368.s001.doc]

Table S1. Current treatment details of patients with TRD

| Treatment | Number of case |
| --- | --- |
| venlafaxine | 5 |
| citalopram | 3 |
| paroxetine hydrochloride | 2 |
| fluoxetine hydrochloride | 1 |
| mirtazapine | 2 |
| amitriptyline | 2 |
| amitriptyline hydrochloride + sodium valproate | 1 |
| amitriptyline + risperidone | 1 |
| paroxetine hydrochloride + quetiapine | 1 |
| venlafaxine + lithium carbonate | 2 |
| imipramine hydrochloride + sodium valproate | 1 |
| unavailable | 2 |

All patients with TSD were treatment-naive. None of the patients with TRD receiving combination treatments had schizoaffective disorder, manic episode or co-morbidity with any Axis II disorders. Combination treatments were being applied because of their claimed efficacy for treatment resistant depression (Bschor, 2010; Little, 2009; Mahmoud et al., 2007).

**References**

Bschor, T., 2010. Therapy-resistant depression. Expert Rev Neurother 10, 77-86.

Little, A., 2009. Treatment-resistant depression. Am Fam Physician 80, 167-172.

Mahmoud, R.A., Pandina, G.J., Turkoz, I., Kosik-Gonzalez, C., Canuso, C.M., Kujawa, M.J., Gharabawi-Garibaldi, G.M., 2007. Risperidone for treatment-refractory major depressive disorder: a randomized trial. Ann Intern Med 147, 593-602.
