# Supplementary material for: Decreased Interhemispheric Coordination in Treatment-Resistant Depression: A Resting-State fMRI Study
Source: PLoS One. 2013 Aug 2;8(8):e71368. doi: 10.1371/journal.pone.0071368 (PMC3732240; doi:10.1371/journal.pone.0071368)
Supplement: Table S2 — The number of episodes of patients with TRD. (DOC) [file pone.0071368.s002.doc]

Table S2. The number of episodes of patients with TRD

| Number of episodes | Number of case |
| --- | --- |
| 1 | 16 |
| 2 | 4 |
| 3 | 3 |

All patients with TSD were at their first episode.
