# Supplementary material for: Decreased Interhemispheric Coordination in Treatment-Resistant Depression: A Resting-State fMRI Study
Source: PLoS One. 2013 Aug 2;8(8):e71368. doi: 10.1371/journal.pone.0071368 (PMC3732240; doi:10.1371/journal.pone.0071368)
Supplement: Table S3 — Significant VMHC differences between pooled patients and HS (p<0.005, GRF correction). (DOC) [file pone.0071368.s003.doc]

Table S3. Significant VMHC differences between pooled patients and HS (*p*<0.005, GRF correction)

| Cluster location | Peak (MNI) | | | Cluster size | *T* value |
| --- | --- | --- | --- | --- | --- |
|  | x | y | z |  |  |
| Patients < HS |  |  |  |  |  |
| Postcentral Gyrus | ±39 | -33 | 57 | 33 | -3.6045 |

VMHC = voxel-mirrored homotopic connectivity

HS = healthy subjects
