# Supplementary material for: Decreased Interhemispheric Coordination in Treatment-Resistant Depression: A Resting-State fMRI Study
Source: PLoS One. 2013 Aug 2;8(8):e71368. doi: 10.1371/journal.pone.0071368 (PMC3732240; doi:10.1371/journal.pone.0071368)
Supplement: Table S4 — Significant VMHC differences between groups (uncorrected voxel-wise threshold of p<0.005). (DOC) [file pone.0071368.s004.doc]

Table S4. Significant VMHC differences between groups (uncorrected voxel-wise threshold of *p*<0.005)

| Cluster location | Peak (MNI) | | | Cluster size | *T* value |
| --- | --- | --- | --- | --- | --- |
|  | x | y | z |  |  |
| TRD < TSD |  |  |  |  |  |
| Calcarine Cortex | ±18 | -69 | 6 | 66 | -3.9644 |
| Fusiform Gyrus | ±33 | -75 | -15 | 268 | -4.8120 |
| Middle Occipital Gyrus | ±36 | -90 | 12 | 48 | -3.4144 |
| Precentral Gyrus | ±57 | 0 | 33 | 62 | -4.4508 |
| Precentral Gyrus | ±45 | -9 | 54 | 46 | -4.0766 |
| Superior Temporal Gyrus | ±51 | -15 | -3 | 63 | -3.4162 |
| Hippocampus | ±24 | -33 | -3 | 110 | -4.0928 |
| Superior Frontal Gyrus | ±21 | 66 | -6 | 23 | -3.2365 |
| Middle Temporal Gyrus | ±48 | -54 | 6 | 68 | -4.0515 |
| Insula | ±36 | -24 | 15 | 25 | -2.7467 |
| Precuneus | ±24 | -72 | 30 | 60 | -3.4555 |
| Middle Cingulum | ±9 | -39 | 45 | 28 | -4.1867 |
| TRD < HS |  |  |  |  |  |
| Calcarine Cortex | ±15 | -72 | 12 | 858 | -3.8629 |
| Cerebelum_Crus1 | ±12 | -84 | -24 | 29 | -3.2923 |
| Fusiform Gyrus | ±30 | -66 | -12 | 29 | -2.9372 |
| Superior Temporal Gyrus | ±51 | -15 | 0 | 20 | -2.8788 |
| Superior Temporal Gyrus | ±63 | -36 | 12 | 34 | -3.5617 |
| Precentral Gyrus | ±60 | 0 | 36 | 50 | -3.2876 |
| Precentral Gyrus | ±30 | 0 | 45 | 71 | -3.1254 |
| Middle Frontal Gyrus | ±45 | 27 | 30 | 62 | -3.6915 |
| Postcentral Gyrus | ±45 | -30 | 33 | 111 | -2.9500 |
| TSD < HS |  |  |  |  |  |
| Postcentral Gyrus | ±48 | -30 | 57 | 26 | -3.0453 |

VMHC = voxel-mirrored homotopic connectivity

TRD = treatment-resistant depression

TSD = treatment-sensitive depression

HS = healthy subjects
